# Comparative Outcomes of Monopolar and Bipolar Transurethral Resection of the Prostate: An Institutional Perspective

**DOI:** 10.7759/cureus.67947

**Published:** 2024-08-27

**Authors:** Mahendra Wante, Mathew John Mathai, Varun Shetty

**Affiliations:** 1 Department of General Surgery, Dr. D. Y. Patil Medical College, Hospital and Research Centre, Dr. D. Y. Patil Vidyapeeth (Deemed to be University), Pune, IND

**Keywords:** prostate volume, q max, hyponatremia, bhp, turp

## Abstract

Objective

This prospective comparative study aimed to highlight and compare two types of transurethral resection of the prostate (TURP), namely M-TURP (monopolar) and B-TURP (bipolar), in the endoscopic management of benign prostatic hyperplasia (BPH).

Methods and materials

This research was conducted between 2022 and 2023 at a tertiary care health center. Included in the investigation were 100 consenting study participants undergoing M-TURP and B-TURP at our center. All referred patients presenting with clinical, ultrasound, or uroflowmetry features of BPH and those with failed attempts at medical management were included in the study. Patients with carcinoma of the prostate were excluded from the study. Post-operatively, the endpoints for comparison included maximal urinary flow rate (Qmax), prostate volume, duration of hospital stay, duration of catheterization, drop in serum sodium concentration, and drop in hemoglobin levels. Descriptive statistics were computed to delineate the study sample. After the completion of data collection, data analysis was performed using SPSS for Windows, Version 16.0 (Released 2007; SPSS Inc., Chicago, IL, USA), and the correlations sought were achieved using the Chi-square test of significance.

Results

The peak incidence of BPH was seen in the sixth decade of life: the M-TURP group was 65.16 ± 7.07 years (mean ± standard deviation), while that in the B-TURP group was 62.32 ± 8.16 years (mean ± standard deviation). Nine percent of the study participants did not show any comorbidities. The most frequent symptom of BPH at presentation was a poor urinary stream (78%, n = 100), followed by nocturia (67%, n = 100). In our study, patients undergoing M-TURP had a mean serum prostate-specific antigen (PSA) level of 4.31 ± 1.03 ng/mL, while patients undergoing B-TURP had a mean serum PSA of 4.24 ± 0.99 ng/mL (p = 0.820; p > 0.05). The study found that patients undergoing M-TURP had a mean prostate size of 35.04 ± 3.57 cc, while those undergoing B-TURP had a mean prostate size of 35.72 ± 3.22 cc (p = 0.765). For the B-TURP group, the mean decrease in postoperative serum sodium concentration was 4.3 mEq/L, while for the M-TURP group, it was 6.4 mEq/L (p = 0.903). In the M-TURP group, there were three cases of transurethral resection (TUR) syndrome, while the B-TURP group had only one case.

Conclusion

BPH is a common problem affecting the quality of life of several male patients. Both M-TURP and B-TURP are comparable in their efficacy in treating BPH, with the exception of a higher incidence of hyponatremia and TUR syndrome in the M-TURP group.

## Introduction

Benign prostatic hyperplasia (BPH) represents a relatively common encounter for physicians, general surgeons, and urologists. It is a benign pathology affecting men, with an increasing incidence relative to the patient’s age [1]. Speakman et al. demonstrated that the majority of cases with BPH presented in the age group of 45-49 years, followed by another peak above 80 years of age [2].

The predominant presentation of BPH is that of an elderly male complaining of progressively increasing lower urinary tract symptoms (LUTS) [3]. A wide plethora of treatment modalities are available to combat BPH, ranging from a more conservative approach to surgical management in the form of endoscopic or open surgery.

Medical management is prescribed initially in the majority of cases. However, over time, the benefit provided by these drugs becomes limited owing to the progressive nature of the illness, and the need for a more definitive approach arises [4,5]. Transurethral resection of the prostate (TURP), since its introduction in the 1930s, has become the most commonly employed modality in present times. This increase in its popularity can be attributed to its shorter hospitalization time, minimal morbidity, and low mortality rate [6,7].

Monopolar TURP (M-TURP) and bipolar TURP (B-TURP) represent two variants of the procedure, of which M-TURP is the current gold standard [8]. They differ in terms of the irrigating fluid utilized: distilled water or glycine in M-TURP and isotonic saline in B-TURP [9,10]. This variation has demonstrated a significantly lower incidence of dilutional hyponatremia and transurethral resection (TUR) syndrome in the bipolar group, increasing its preference, especially when dealing with larger glands.

Using this study, we attempted to highlight the differences in intra-operative as well as post-operative parameters of M-TURP and B-TURP, to provide a better understanding of their merits and demerits.

## Materials and methods

This prospective observational research was conducted between 2022 and 2023 at a tertiary care health center. Included in the investigation were 100 consenting study participants undergoing M-TURP and B-TURP for BPH at our center. Institutional Ethics Sub-Committee approval was obtained prior to beginning the study (I.E.S.C/74/2022). The study was initiated only after obtaining informed consent from all the participants. All referred patients presenting with clinical, ultrasound, or uroflowmetry features of BPH, and those with failed attempts at medical management were included in the study. The medical management included the administration of selective beta-blockers, such as tamsulosin, for three weeks.

The diagnostic criteria employed for inclusion in the study were based on the presence of clinical, ultrasound, and uroflowmetry features of BPH. These included clinical (symptoms of bladder outlet obstruction, LUTS), ultrasound (prostate volume exceeding 30 g), and uroflowmetry (peak urinary flow rate <12 mL/s). Patients with diagnosed carcinoma of the prostate, neurogenic bladder, and those with failed attempts at medical management were excluded from the study. The patients were then divided into two groups: M-TURP and B-TURP. The first patient was allotted to the M-TURP group, and the following patients were alternately allotted to both groups (n = 50 in each group).

All the included participants in each group were evaluated thoroughly by means of a detailed clinical examination and biochemical evaluation (including complete blood count, serum electrolytes, renal and liver function tests, coagulation profile, and serum prostate-specific antigen (PSA) levels), and the findings were logged in dedicated proformas created for this research. In each group, the pre-operative maximal urinary flow rate (Qmax) was documented, and a comparison was made to determine the severity of BPH. In addition, ultrasound parameters (pre-void volume, post-void volume, and prostate size) were documented and later compared with their post-operative counterparts to assess the effectiveness of the procedure. The patients were then subjected to the endoscopic procedure, i.e., M-TURP or B-TURP.

We performed the M-TURP procedure using a 26-French Karl Storz resectoscope and a 1.5% glycine solution as the irrigation medium. The procedure utilized a wire loop that carried an electric current in a single direction to resect prostatic tissue. We used a grounding pad and irrigation with a non-conducting medium, such as a 1.5% glycine solution, to prevent the current from disrupting nearby tissues. A power setting of 120 W was employed to resect the gland and achieve hemostasis. B-TURP was performed using a 26-Fr Olympus resectoscope, with normal saline used for irrigation. We used an energy current setting of 200 W for the cutting process and 120 W for coagulation. The bidirectional current flow enhances hemostasis; additionally, it obviates the necessity for an electrolyte-free irrigation solution, such as 1.5% glycine in M-TURP. The same surgical team performed both procedures. Depending on the patient's general condition, the surgical team performed the procedure under either spinal or general anesthesia.

The parameters recorded intra-operatively (operative time and blood loss) and post-operatively (drop in hemoglobin/Hb, blood transfusion requirement, post-procedure Qmax, prostate size/volume, sodium levels, duration of hospital stay, and duration of catheterization) were documented in the proforma mentioned above. The occurrence of complications, such as hemorrhage and TUR syndrome, was also documented. The recorded parameters from each group, as mentioned above, were compared using a Chi-square test of statistical significance to establish the effectiveness of both procedures.

Descriptive statistics, like mean and standard deviation, were computed to delineate the study sample. After the completion of data collection, data analysis was conducted using SPSS for Windows, Version 16.0 (Released 2007; SPSS Inc., Chicago, IL, USA). Differences between the means of the two groups were compared using an independent samples t-test. For all the tests, a p-value <0.05 indicates statistical significance.

## Results

In our study, the greatest incidence of BPH occurred around the sixth decade of life. The mean age in the M-TURP group was 65.16 ± 7.07 years (mean ± standard deviation), while in the B-TURP group, it was 62.32 ± 8.16 years (mean ± standard deviation), according to Table 1.

**Table 1:** Age-wise distribution of patients in both groups (in years)

Age (years)	n (%)
41-50	13 (13%)
51-60	26 (26%)
61-70	46 (46%)
71-80	15 (15%)
Total	100

A total of 100 patients underwent TURP over a two-year period, with 50 undergoing M-TURP (n = 50) and 50 undergoing B-TURP (n = 50). Nine percent (n = 100) of the trial participants had no comorbidities, but 20% (n = 100) had diabetes mellitus, and 17% (n = 100) had hypertension, both of which hampered the patients' recovery.

The most common symptom of BPH at presentation was a weak urine stream (78% of 100), followed by nocturia (67%). According to our findings, 74% of the patients had serum PSA levels of more than 4 ng/mL. Patients receiving M-TURP had a mean preoperative PSA level of 4.31 ± 1.03 ng/mL, which was identical to those undergoing B-TURP (4.24 ± 0.99 ng/mL). However, there was no statistically significant change in the measured values after surgery (p = 0.820; p > 0.05).

The study discovered that individuals undergoing M-TURP had a mean prostate size of 35.04 ± 3.57 cc, while those undergoing B-TURP had a mean prostate size of 35.72 ± 3.22 cc (p = 0.765; p > 0.05). In our study, patients receiving B-TURP had a mean preoperative Qmax of 9.73 ± 1.06 mL/s (mean ± standard deviation), while those undergoing M-TURP had a mean preoperative Qmax of 9.51 ± 1.26 mL/s (mean ± standard deviation), resulting in a statistically insignificant difference (p = 0.148).

The mean decrease in serum sodium concentration after surgery for the B-TURP group was 4.3 ± 1.2 mEq/L, while for the M-TURP group, it was 6.4 ± 1.4 mEq/L (p = 0.903). The M-TURP group had three cases of TUR syndrome, whereas the B-TURP group had only one. The M-TURP group had an average hemoglobin drop of 0.71 ± 0.08 g/dL, while the B-TURP group had a significantly lower decline of 0.54 ± 0.02 g/dL (p = 0.008; p < 0.05).

The M-TURP group had a mean postoperative catheter duration of 54.5 ± 7.62 hours, while the B-TURP group had a mean of 52.25 ± 7.88 hours. This difference was statistically insignificant (p = 0.672). In our study, the M-TURP group had an average hospitalization stay of 3.04 ± 0.06 days, compared to 2.96 ± 0.02 days in the B-TURP group (p = 0.543). The B-TURP group had a substantially longer mean operative time compared to the M-TURP group (41.74 ± 5.15 minutes vs. 44.04 ± 4.02 minutes; p = 0.04). The above-mentioned observations are presented in tabular form in Table 2.

**Table 2:** Comparison of parameters in the M-TURP and B-TURP group Values represent the mean and standard deviation; the test used was an independent samples t-test. A p-value <0.05 indicates statistical significance. M-TURP: Monopolar-transurethral resection of the prostate; B-TURP: Bipolar-transurethral resection of the prostate; TUR: Transurethral resection

Parameter	M-TURP group (n = 50)	B-TURP group (n = 50)	p-value
Mean age (in years)	65.16 ± 7.07 years	62.32 ± 8.16 years	0.054
Mean pre-operative prostate volume (in cc)	35.04 ± 3.57 cc	35.72 ± 3.22 cc	0.765
Mean pre-operative maximal urinary flow rate i.e., Qmax (in mL/s)	9.51 ± 1.26 mL/s	9.73 ± 1.06 mL/s	0.148
Mean drop in Hb (in g/dL)	0.71 ± 0.08 g/dL	0.54 ± 0.02 g/dL	0.008
Mean change in sodium levels (in mmol/L)	6.4 ± 1.4 mEq/L	4.3 ± 1.2 mEq/L	0.903
TUR syndrome	3	1	-
Mean postoperative catheter duration (in hours)	54.5 ± 7.62 hours	52.25 ± 7.88 hours	0.672
Duration of hospital stay (in days)	3.04 ± 0.06 days	2.96 ± 0.02 days	0.543

## Discussion

BPH is one of the most common causes of bladder outlet obstruction encountered by surgeons around the world [11]. This pathology not only manifests as numerous troublesome symptoms for the patient but also dramatically affects their quality of life. Since its introduction in 1930, TURP has stood at the forefront of surgical modalities available against BPH [10,11]. The reason for this procedure having stood the test of time lies in its low morbidity and high success rate. M-TURP is the well-established variant of the procedure that is still the gold standard for BPH even today. However, like with any procedure, M-TURP also carries its own set of complications, such as dilutional hyponatremia, TUR syndrome, and hemorrhage. These setbacks account for 11.1% of cases, according to the findings of a multicenter study involving 10,654 male participants with BPH [12].

In recent years, innovative approaches, such as the use of B-TURP, have challenged the traditional M-TURP. When a bipolar generator is used, both the active and return electrodes are integrated within the device, as opposed to the circuit used in M-TURP [13]. The most notable advantage is the ability to use regular saline as the irrigation fluid, rather than traditional distilled water or glycine. This reduces the danger of electrolyte imbalances caused by systemic uptake of the irrigation fluid, which can result in conditions such as TUR syndrome [14].

In keeping with Madduri et al., the mean age of onset in our investigation was 65.16 ± 7.07 years (mean ± standard deviation) in the monopolar group and 62.32 ± 8.16 years (mean ± standard deviation) in the bipolar group [15]. The observed behavior can be related to the typical presentation of BPH at a later age, after 60 years. In the current investigation, the pre-operative Qmax in the monopolar group was 9.86 ± 0.93 mL/s, compared to 9.53 ± 1.26 mL/s in the bipolar group. However, the change was not statistically significant. These findings were consistent with those of Madduri et al., who found that both groups' pre-operative Qmax values fell within the same range [15]. Yoon et al. found similar pre-operative Qmax values between the two groups (p = 0.866) [16]. In contrast, Kong et al. reported a significant difference in pre-operative Qmax values, with the bipolar group having a lower mean value (Qmax: 7.81 ± 2.51 mL/s) [17].

Our findings revealed a significant fall in hemoglobin (Hb) levels in the monopolar group (p = 0.008). Madduri et al. found that the average drop in Hb for the M-TURP group was 1.57 ± 0.71 g/dL, compared to 1.75 ± 0.77 g/dL for the B-TURP group, which was statistically insignificant (p = 0.28) [15]. In contrast, Giulianelli et al. found that the M-TURP group had a significantly lower Hb level (14.52 to 10.4 mg/dL) than the B-TURP group (14.88 to 13.6 mg/dL) [18]. Our study found that, even though the glands operated on using the B-TURP technique were equal in size, the ensuing fall in Hb was less severe than that of the M-TURP group (Table 3).

**Table 3:** Comparison of the drop in hemoglobin post-surgery in both groups, i.e., M-TURP and B-TURP, as per existing literature M-TURP: Monopolar-transurethral resection of the prostate; B-TURP: Bipolar-transurethral resection of the prostate

Study	Monopolar	Bipolar	Level of significance (p-value)
Yoon et al. [16]	0.62 g/dL	0.67 g/dL	Not significant (0.23)
Kong et al. [17]	1.8 g/dL	0.6 g/dL	Not available (NA)
Chen et al. [19]	1.1 g/dL	1.6 g/dL	Not available (NA)
Skolarikos et al. [20]	1.1 g/dL	0.9 g/dL	Not significant (0.08)
Fagerström et al. [21]	9.59 g/dL	5.54 g/dL	Significant (0.04)

Our findings showed that the total degree of hyponatremia experienced post-operatively was comparable between the two groups (p = 0.908). However, M-TURP resulted in a greater decline in sodium levels (6.4 ± 5.1 mmol/L) compared to B-TURP (4.3 ± 3.2 mmol/L). As a result, TUR syndrome occurred in three cases of M-TURP, compared to one in the B-TURP group. According to Madduri et al., the M-TURP group had a significantly lower serum sodium concentration (p = 0.001) than the B-TURP group [15]. They reiterated that the M-TURP group had a higher risk of developing TUR syndrome (relative risk: 0.17). The suggested pathophysiology responsible for the formation of TUR syndrome after TURP is excessive absorption of the irrigation fluid via the peri-prostatic pathways, resulting in hypervolemia and concomitant dilutional iso-osmolar hyponatremia (Table 4).

**Table 4:** Comparison of the occurrence of TUR syndrome post-surgery in both groups, i.e., M-TURP and B-TURP, as per existing literature M-TURP: Monopolar-transurethral resection of the prostate; B-TURP: Bipolar-transurethral resection of the prostate

Study	M-TURP	B-TURP
Yoon et al. [16]	9	5
Chen et al. [19]	3	0
Skolarikos et al. [20]	4	1

The average hospitalization length for patients who received M-TURP was 3.04 ± 0.06 days, whereas for the B-TURP group, it was 2.96 ± 0.02 days (p = 0.543). The B-TURP group had a somewhat shorter average catheter duration than the M-TURP group.

The study has drawbacks, including a small sample size, a lack of bias-prevention methods, and a short follow-up period. Nonetheless, we want to continue our research by combining a larger study cohort and generating more thorough data that can help surgeons rigorously select the optimal therapy according to the circumstances.

## Conclusions

BPH represents a significant deterrent to the quality of life in males, owing to the symptoms as well as the financial burden it carries. Various treatment modalities have emerged over the years, ranging from medical management and endoscopic procedures (TURP) to open surgery, though with limited indications. An in-depth understanding of these modalities, including their merits and demerits, can aid a surgeon or physician in making a calculated decision specifically tailored to the patient’s condition, allowing for a better clinical outcome. By means of this investigation, we have attempted to highlight one aspect among this wide array of procedures, i.e., TURP, and shed light on its variations and prospects, thus adding to the ever-expanding sea of knowledge.
